# Protein Kinase C-Independent Inhibition of Organic Cation Transporter 1 Activity by the Bisindolylmaleimide Ro 31-8220

**DOI:** 10.1371/journal.pone.0144667

**Published:** 2015-12-10

**Authors:** Abdullah Mayati, Arnaud Bruyere, Amélie Moreau, Elodie Jouan, Claire Denizot, Yannick Parmentier, Olivier Fardel

**Affiliations:** 1 Institut de Recherches en Santé, Environnement et Travail (IRSET), UMR INSERM U1085, Faculté de Pharmacie, 2 Avenue du Pr Léon Bernard, 35043, Rennes, France; 2 Centre de Pharmacocinétique, Technologie Servier, 25–27 rue Eugène Vignat, 45000, Orléans, France; 3 Pôle Biologie, Centre Hospitalier Universitaire, 2 rue Henri Le Guilloux, 35033, Rennes, France; Emory University, UNITED STATES

## Abstract

Ro 31–8220 is a potent protein kinase C (PKC) inhibitor belonging to the chemical class of bisindolylmaleimides (BIMs). Various PKC-independent effects of Ro 31–8220 have however been demonstrated, including inhibition of the ATP-binding cassette drug transporter breast cancer resistance protein. In the present study, we reported that the BIM also blocks activity of the solute carrier organic cation transporter (OCT) 1, involved in uptake of marketed drugs in the liver, in a PKC-independent manner. Ro 31–8220, in contrast to other pan-PKC inhibitors such as staurosporine and chelerythrine, was thus shown to *cis*-inhibit uptake of the reference OCT1 substrate tetraethylammonium in OCT1-transfected HEK293 cells in a concentration-dependent manner (IC_50_ = 0.18 μM) and without altering membrane expression of OCT1. This blockage of OCT1 was also observed in human hepatic HepaRG cells that constitutionally express OCT1. It likely occurred through a mixed mechanism of inhibition. Ro 31–8220 additionally *trans*-inhibited TEA uptake in OCT1-transfected HEK293 cells, which likely discards a transport of Ro 31–8220 by OCT1. Besides Ro 31–8220, 7 additional BIMs, including the PKC inhibitor LY 333531, inhibited OCT1 activity, whereas 4 other BIMs were without effect. *In silico* analysis of structure-activity relationships next revealed that various molecular descriptors, especially 3D-WHIM descriptors related to total size, correspond to key physico-chemical parameters for inhibition of OCT1 activity by BIMs. In addition to activity of OCT1, Ro 31–8220 inhibited those of other organic cation transporters such as multidrug and toxin extrusion protein (MATE) 1 and MATE2-K, whereas, by contrast, it stimulated that of OCT2. Taken together, these data extend the nature of cellular off-targets of the BIM Ro 31–8220 to OCT1 and other organic cation transporters, which has likely to be kept in mind when using Ro 31–8220 and other BIMs as PKC inhibitors in experimental or clinical studies.

## Introduction

Ro 31–8220 is a potent pan-protein kinase C (PKC) inhibitor belonging to the chemical class of bisindolylmaleimides (BIMs), that contains 11 chemicals, numbered from BIM-I to BIM-XI, initially characterized for their putative interaction with PKCs [1]. Ro 31–8220 (also known as BIM-IX) inhibits PKC activity in various types of cells, including platelets and T lymphocytes [2], which is consistent with the fact that this lipophilic chemical is a cell-permeable compound, that most likely enters cells through passive diffusion as well-established for hydrophobic chemicals [3]. It notably blocks activity of classical α, β1, β2 and γ PKC isoforms [4] and is also thought to inhibit novel δ and θ and atypical ι and ζ PKC isoforms [5–10]. Ro 31–8220 has been consequently largely used in experimental studies for investigating PKC implications in various physiological, pathological or pharmacological cellular regulatory ways [11].

Several PKC-independent effects of Ro 31–8220 have however been reported, thus highlighting the lack of specificity of this PKC inhibitor [12]. Ro 31–8220 notably inhibits mitogen-activated protein kinase (MAPK) phosphatase-1 [13], RSK1, RSK2 and RSK3 isoforms of the p90 ribosomal S6 kinase [14], p70 ribosomal S6 kinase [15, 16], CDC2 histone H1 kinase [17] and glycogen synthase kinase-3 [18]. It also activates phosphoinositide phospholipase C and c-Jun N-terminal kinase, induces apoptosis in tumoral cells and blocks voltage-dependent sodium channels in a PKC-independent manner [19–22].

Inhibition of membrane ATP-binding cassette (ABC) drug transporters constitutes another type of off-target effects for Ro 31–8220 and related BIMs. Thus, GF 109203X (also known as BIM-I or Gö 6850) directly inhibits activity of the ABC transporters P-glycoprotein (*ABCB1*) and multidrug resistance-associated protein (MRP) 1 (*ABCC1*) [23, 24], thereby blocking efflux of anticancer drugs mediated by these pumps and consequently reversing drug resistance in tumoral cells. In the same way, activity of breast cancer resistance protein (BCRP/*ABCG2*), an ATP efflux pump also involved in anticancer drug resistance, is blocked by BIMs, including Ro 31–8220 [25]. In the present study, we report that the solute carrier (SLC) organic cation transporter (OCT) 1 (*SLC22A1*), known to play a major role in membrane transport of marketed drugs like metformine in the liver [26] and also recently identified as a high-capacity thiamine transporter that regulates hepatic steatosis [27], is markedly inhibited by Ro 31–8220 in a PKC-independent manner. In addition, Ro 31–8220 interacts with activities of other organic cation transporters, such as those of multidrug and toxin extrusion protein (MATE) 1 (*SLC47A1*) and MATE2-K (*SLC47A2*), which are inhibited, and that of OCT2 (*SLC22A2*), which is unexpectedly stimulated. Such data therefore extend the nature of the cellular off-targets of Ro 31–8220 to SLC transporters and suggest that caution is required when using Ro 31–8220 and related BIMs for studying potential protein kinase-mediated-regulation of drug transporter activity or expression, especially with respect to SLC transporters of organic cations.

## Materials and Methods

### Chemicals

**Fig 1:**
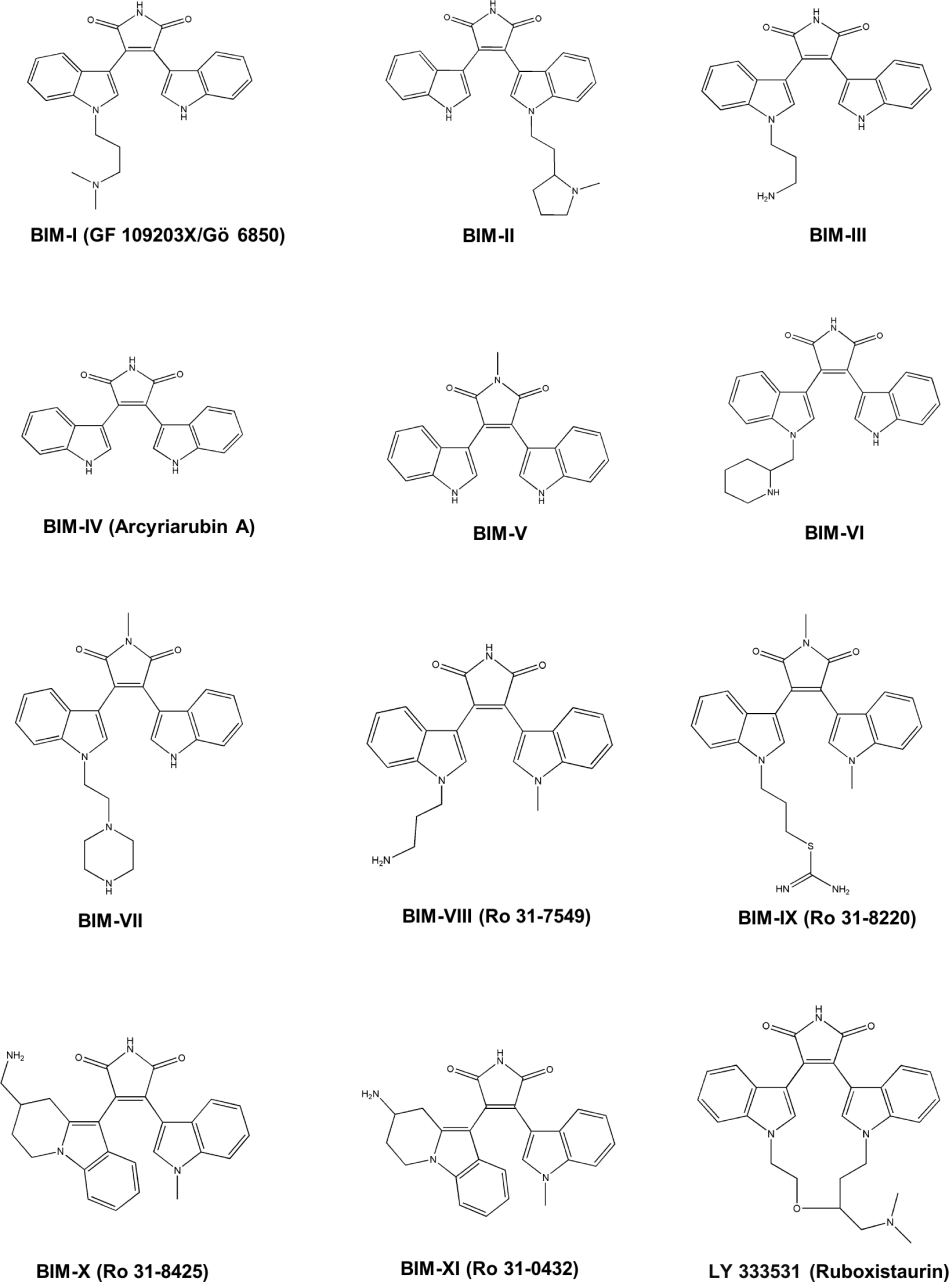
Chemical structures of BIMs.

BIMs I-XI and the PKC inhibitor LY 333531 (also known as ruboxistaurin), that contains a BIM core, were provided by Santa Cruz Biotechnology (Dallas, Texas) and Sigma-Aldrich (Saint-Quentin Fallavier, France). The chemical structures of these BIMs are shown in Fig 1. Verapamil, cyclosporin A, Gö 6983, amitriptyline, U0126, wortmannin, KN62, staurosporine, chelerythrine, tetra-ethylammonium (TEA), 4',6-diamidino-2-phenylindole (DAPI) and phorbol-12-myristate-13-acetate (PMA) were purchased by Sigma-Aldrich (Saint-Quentin Fallavier, France). [1-^14^C]-TEA (sp. act. 2.4 mCi/mmol) and [^3^H(G)] taurocholic acid (sp. act. 1.19 Ci/mmol) were from Perkin-Elmer (Boston, MA). All other chemicals were commercial products of the highest purity available.

### Cell culture

HEK293 cells overexpressing OCT1 (NM_003057) (HEK-OCT1 cells), OCT2 (NM_003058) (HEK-OCT2 cells), MATE1 (NM_018242) (HEK-MATE1 cells), MATE2-K (NM_152908) (HEK-MATE2-K cells) or sodium/taurocholate co-transporting polypeptide (NTCP/*SLC10A1*) (NM_003049) (HEK-NTCP cells) were prepared by transduction of HEK293 cells by a lentiviral pLV-EF1-hOCT1-hPGK-GFP, pLV-EF1-hOCT2-hPGK-GFP, pLV-EF1-hMATE1-hPGK-GFP, pLV-EF1-hMATE2K-hPGK-GFP or pLV-EF1-hNTCP-hPGK-GFP vector, as previously described [28]. Control HEK293 cells (HEK-MOCK cells) were obtained in parallel by transduction of an empty lentiviral PLV-EF1-hPGK-GFP vector. Construction of the lentiviral vectors, production of lentivirus supernatants, transduction of HEK293 cells, cloning and initial characterization of HEK-OCT1, HEK-OCT2, HEK-MATE1, HEK-MATE2-K and HEK-NTCP cells were performed by Vectalys (Labège, France). HEK293 cells were next routinely cultured in DMEM medium supplemented with 10% (vol/vol) fetal calf serum, 100 IU/mL penicillin, 100 μg/mL streptomycin, and 1 μg/mL insulin.

Human highly-differentiated hepatoma HepaRG cells [29], which exhibit functional expression of OCT1 [30], were routinely cultured in Williams' E medium (Life Technologies) supplemented with 10% (vol/vol) fetal calf serum, 100 IU/mL penicillin, 100 μg/mL streptomycin, 5 μg/mL insulin, 2 mM glutamine, and 5 x 10^−5^ M hydrocortisone hemisuccinate. Additional culture for two weeks in the same medium supplemented with 2% (vol/vol) dimethylsulfoxide was performed in order to get a full hepatocytic differentiation of the cells [30].

### Uptake assays

[1-^14^C] TEA and DAPI uptake assays were performed using a transport assay medium consisting of 5.3 mM KCl, 1.1 mM KH_2_PO_4_, 0.8 mM MgSO_4_, 1.8 mM CaCl_2_, 11 mM D-glucose, 10 mM HEPES, and 136 mM NaCl [28]; pH was adjusted to 7.4 value for OCT1, OCT2 and NTCP assays and to 8.4 value for the pH-sensitive MATE1 and MATE2-K assays. Cells were first washed with transport assay buffer, then incubated at 37°C with transport assay buffer containing 40 μM [1-^14^C] TEA (a reference substrate for OCT1, OCT2, MATE1 and MATE2-K), 43.4 nM [^3^H] taurocholate (a reference substrate for NTCP) or 1 μM DAPI (a substrate for OCT1 [31]), in the absence or presence of BIMs or reference inhibitors, *i*.*e*., verapamil for OCT1, MATE1 and MATE2-K, amitriptyline for OCT2, and cyclosporin A for NTCP. The incubation time with substrates was 5 min, that corresponds to the initial linear phase of substrate uptake, notably for OCT1 [28], and is in the range of incubation periods already retained in previous transport studies [32, 33]. Cells were next washed twice with ice-cold phosphate-buffered saline (PBS), and finally lysed in distilled water. DAPI content was next determined using a SpectraMax Gemini SX spectrofluorometer (Molecular Devices, Sunnyvale, CA, USA); excitation and emission wavelengths were 355 and 460 nm, respectively. TEA and taurocholate accumulation were measured by scintillation counting. Data were usually expressed as % of substrate accumulation found in control cells; for OCT1-mediated transport inhibition by BIMs, data were also expressed as % of inhibition of OCT1 activity using the following equation:

%inhibition of OCT1activity=100%-%TEA accumulation(in the presence of BIM)

### *Trans*-stimulation assays

[1-^14^C] TEA trans-stimulation assays were performed in HEK-OCT1 cells as previously described [34]. Cells were first preloaded with 2 mM unlabeled TEA or 10 μM Ro 31–8220 for 60 min at 37°c in the transport assay medium described above. After washing with PBS, cells were re-incubated with 40 μM [1-^14^C] TEA for 5 min at 37°C. Intracellular accumulation of [1-^14^C] TEA was then determined by scintillation counting as reported above.

### Kinetic parameters for Ro 31-8220-mediated modulation of drug transporter activities

Half maximal inhibitory concentrations (IC_50_) for Ro 31-8220-mediated inhibition of OCT1, MATE1 and MATE2-K activities and half maximal effective concentration (EC_50_) for Ro 31-8220-mediated stimulation of OCT2 activity were determined using Prism software (GraphPad Software, La Jolla, CA) through nonlinear regression based on the four-parameter logistic equation, as previously described [35]. Mode of action for Ro 31-8220-mediated inhibition of OCT1-related TEA uptake was evaluated through graphical analysis of Lineweaver-Burke plots of 1/v (v is defined as the specific uptake velocity of TEA) as a function of 1/[TEA], in the presence and absence of the BIM. Non-linear regression according to the Michaelis-Menten equation was finally performed using Prism software in order to fit v as a function of [TEA] in HEK-OCT2 cells, in the absence or presence of Ro 31–8220, and thus to determine kinetic parameters such as K_m_ (Michaelis-Menten constant) and V_max_ (maximum specific uptake) values.

### Molecular descriptor generation

Molecular descriptors were evaluated using the Dragon 6 software (Talete, Milano, Italy) that provides 4885 molecular descriptors that are divided into 29 blocks. These blocks include 3D descriptors such as WHIM (Weighted Holistic Invariant Molecular) descriptors, which contain information about the whole 3D molecular structure in terms of size, shape, symmetry and atom distribution [36]. These 3D-WHIM indices are calculated from x,y,z-coordinates of a 3D structure of the molecule, usually from a spatial conformation of minimum energy, within different weighting schemes in a straightforward manner and represent a very general approach to describe molecules in a unitary conceptual framework. BIMs, initially expressed in SMILES format, were converted to 3D format using the MarvinView software (ChemAxon, Budapest, Hungary) before processing by Dragon 6 to obtain 0D constitutional, 1D structural, 2D topological and 3D geometrical descriptors.

### Immunolocalisation

Immunofluorescence analyses were performed as previously described [28]. Briefly, HEK-OCT1 cells were first fixed in ice-cold acetone for 10 min. Cells were next incubated for 2 h with mouse monoclonal primary antibody directed against OCT1 (Abcam, Cambridge, UK). After washing, an AlexaFluor 488-labeled goat secondary antibody directed against mouse IgG (Invitrogen) was added for 1 h, and nuclei were subsequently stained with 4,6-diamidino-2-phenylindole. Immunofluorescence images were finally detected with an inverted laser scanning confocal Leica DMRXa microscope (Leica, Rueil Malmaison, France).

### Western-blot analysis

Total cellular extracts were prepared as previously reported [37]. Protein lysates (50 μg) were then separated on polyacrylamide gels and electrophoretically transferred onto nitrocellulose membranes (Bio-Rad, Marne la Coquette, France). After blocking with Tris-buffered saline containing 4% bovine serum albumin and 0.1% Tween 20, membranes were incubated overnight at 4°C with primary antibodies against phospho-extracellular signal-regulated kinase (ERK) and total ERK (Cell Signaling, Danvers, MA). After washing, membranes were next incubated for 1 h at room temperature with horseradish peroxidase-conjugated secondary antibodies (Dako A/S, Glostrup, Denmark). Immunolabeled proteins were finally visualized by chemiluminescence.

### Statistical analysis

Experimental data were routinely expressed as means ± SEM. Statistical analysis was performed using the Student's *t*-test, ANOVA followed by a Dunnett's post-hoc test or F-test using Prism software; the criterion of significance was p < 0.05. Correlation between molecular descriptor indexes and % of OCT1 activity inhibition by chemicals was initially done with Dragon 6 software through Pearson correlation, using a cut-off for Pearson’s correlation coefficient of r > 0.8 (positive correlation) or r < -0.8 (negative correlation), as recommended by the software user’s manual. P-values for Pearson correlations, as well as linear regressions for molecular descriptors exhibiting a high level of correlation with OCT1 activity inhibition (|r| > 0.9), were next determined using Prism software after confirmation of normality of data distribution by D'Agostino and Pearson omnibus normality test.

## Results

### PKC-independent inhibition of OCT1 activity by Ro 31–8220

**Fig 2:**
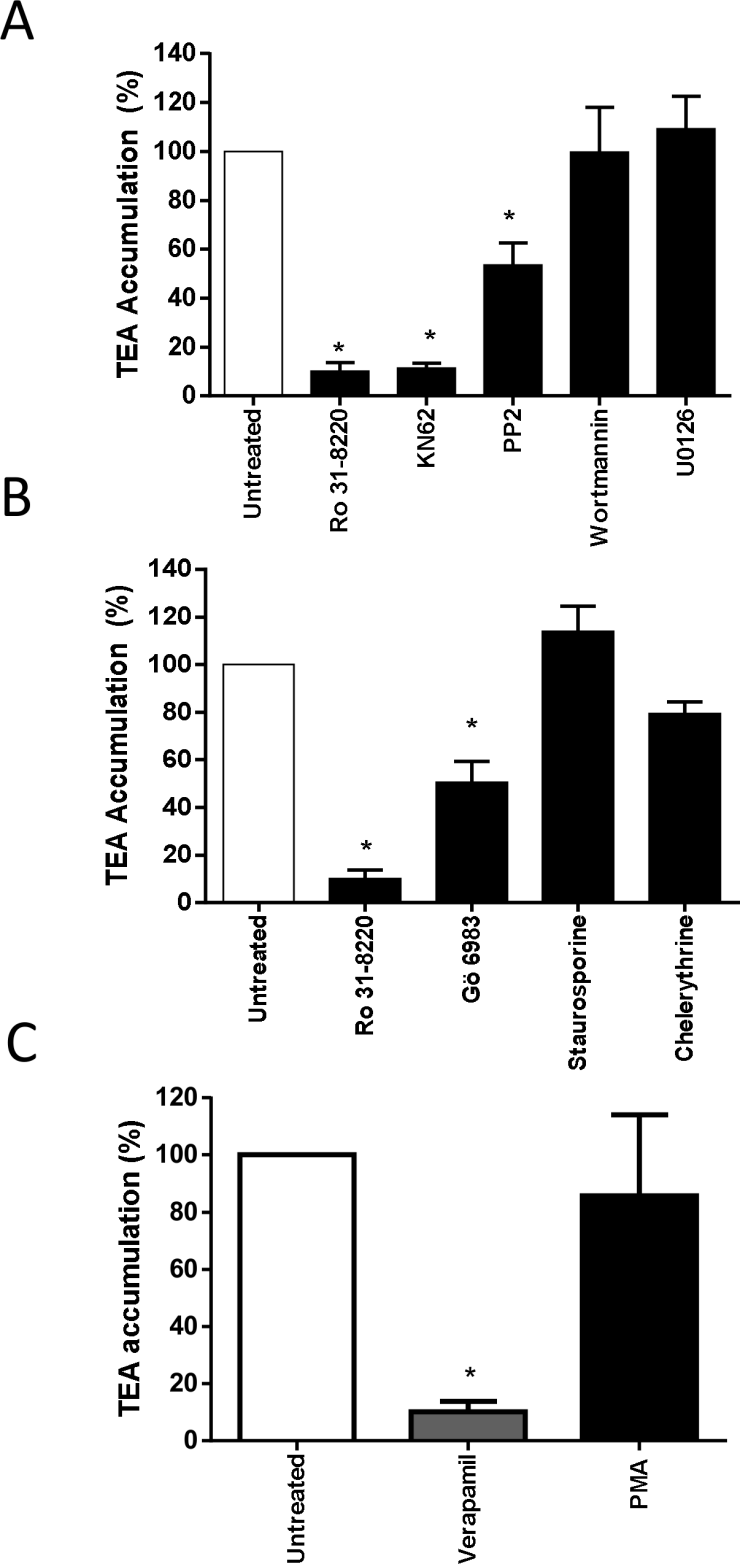
Effect of PKC and other signaling pathways inhibition on OCT1 activity. HEK-OCT1 cells were exposed, or not, for 1 h to (A) 2 μM Ro 31–8220, 20 μM KN62, 20 μM PP2, 200 nM wortmannin or 5 μM U0126, (B) 2 μM Ro 31–8220, 5 μM Gö 6983, 1 μM staurosporine or 50 μM chelerythrine or (C) 100 nM PMA. Cells were next incubated with 40 μM [^14^C]-TEA for 5 min at 37°C in the (A-C) absence or (C) presence of 50 μM verapamil, used here as a reference OCT1 inhibitor. After washing with ice-cold PBS, intracellular accumulation of TEA was determined by scintillation counting. Data are expressed as % of accumulation of TEA found in untreated control cells, set at 100%, and are the means ± SEM of at least three independent experiments. *, p < 0.05 when compared with untreated cells (ANOVA followed by Dunnett's post-hoc test).

Because PKCs have been hypothesized to contribute to regulation of membrane transport of organic cations [38, 39], the effect of Ro 31-8220-mediated inhibition of basal PKC activity on OCT1 activity was initially investigated. For this purpose, we used HEK-OCT1 cells that exhibited a high level of verapamil-inhibitable uptake of the reference OCT1 substrate TEA when compared to control HEK-MOCK cells (S1A Fig), indicating that OCT1 was fully functional in these OCT1-transfected cells, as previously described [28]. As shown in Fig 2A, pre-treatment of HEK-OCT1 cells for 1 h by Ro 31–8220, used at a 2 μM concentration fully active against PKCs [4], resulted in a marked inhibition of TEA influx in HEK-OCT1 cells. By contrast, pre-treatment by Ro 31–8220 of HEK-NTCP cells failed to alter intracellular uptake of the NTCP substrate taurocholate (S2 Fig), thus discarding any unspecific general inhibitory effect of the BIM towards SLC transporter activity. OCT1 activity in HEK-OCT1 cells was also inhibited by pre-treatment with the calcium/calmodulin-dependent protein kinase (CaMK) inhibitor KN62 and by the Src kinase inhibitor PP2, used here as positive controls of OCT1 modulation because the calcium/CaMK pathway as well as the Src kinase Lck have been already shown to be involved in OCT1 activity regulation [40]. By contrast, pre-treatment with wortmannin, a chemical inhibitor of phosphatidylinositol-4,5-bisphosphate 3-kinase, failed to alter TEA accumulation in HEK-OCT1 cells (Fig 2A), which agrees with previous data [40]. The MAPK inhibitor U0126 similarly failed to impair OCT1 activity (Fig 2A).

To determine whether down-regulation of OCT1 activity may also occur in response to PKC inhibition by chemical inhibitors distinct from Ro 31–8220, we next pre-treated HEK-OCT1 cells by various structurally-divergent pan-PKC inhibitors, *i*.*e*., Gö 6983, staurosporine and chelerythrine, before measuring TEA influx. As shown in Fig 2B, both staurosporine and chelerythrine failed to reduce OCT1 activity, whereas Gö 6983 was only partially active. Staurosporine was however fully active as a potent pan-PKC inhibitor in HEK-OCT1 cells because it fully antagonized the known PKC-mediated activation of the MAPK ERK by the phorbol ester PMA in HEK cells [41] (S3 Fig). This most likely indicates that the inhibitory effect of Ro 31–8220 on OCT1 activity was not related to PKC inhibition. Moreover, activation of PKCs by PMA treatment failed to modify TEA accumulation in HEK-OCT1 cells (Fig 2C), which also does not argue in favor of a regulation of OCT1 activity by PKCs.

### Direct *cis*-inhibition of OCT1 activity by Ro 31–8220

**Fig 3:**
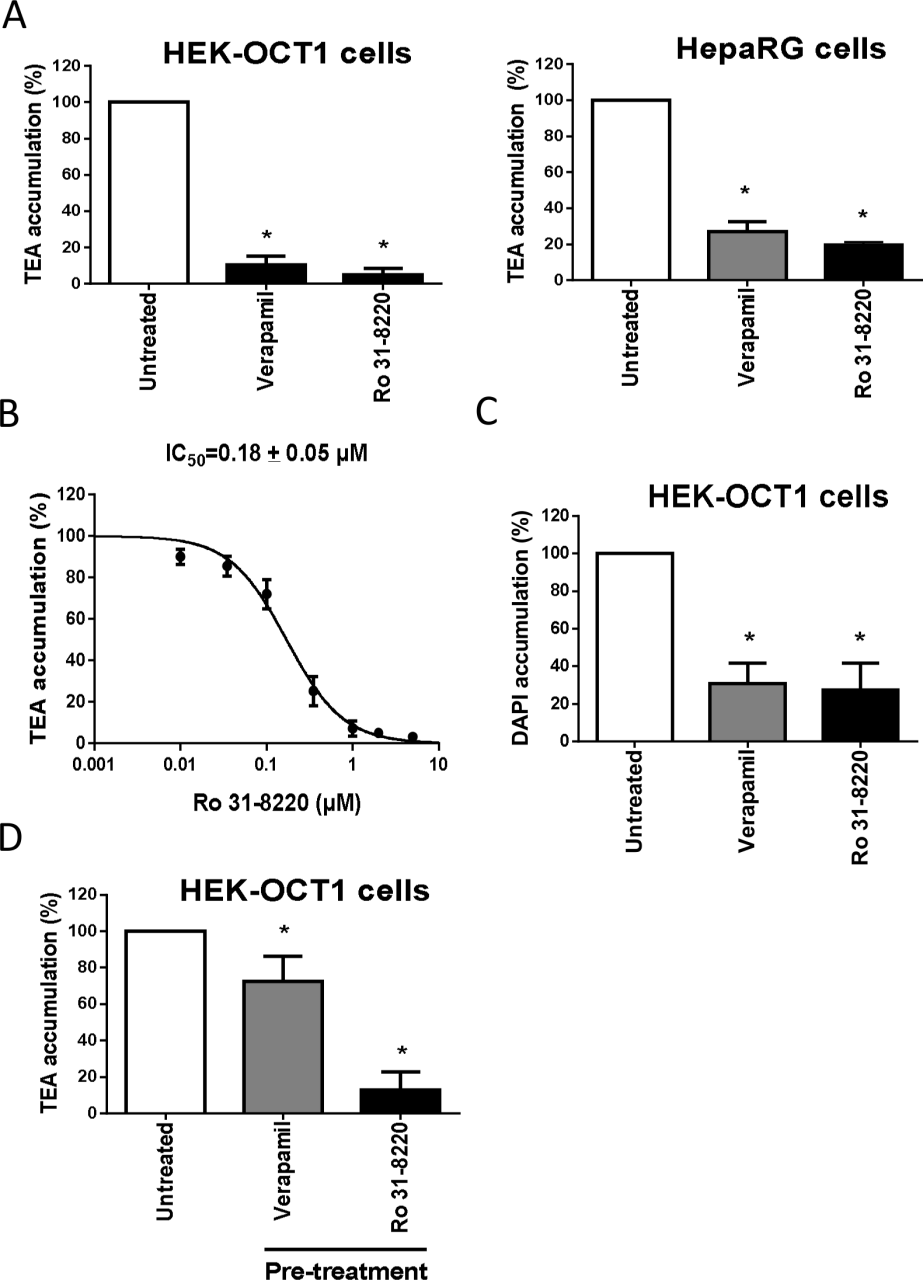
Direct cis-inhibition of OCT1 activity by Ro 31–8220. (A) HEK-OCT1 and HepaRG cells were incubated with 40 μM [^14^C]-TEA for 5 min at 37°C, in the presence or absence of 50 μM verapamil or 2 μM Ro 31–8220. After washing with ice-cold PBS, intracellular accumulation of TEA was determined by scintillation counting. Data are expressed as % of accumulation of TEA found in control cells, set at 100%, and are the means ± SEM of at least three independent experiments. *, p < 0.05 when compared with untreated cells (ANOVA followed by Dunnett's post-hoc test). (B) HEK-OCT1 cells were incubated with 40 μM [^14^C]-TEA for 5 min at 37°C, in the presence of various concentrations of Ro 31–8220 (from 0 to 10 μM). After washing with ice-cold PBS, intracellular accumulation of TEA was determined by scintillation counting. Data are expressed as % of accumulation of TEA found in control cells, set at 100%, and are the means ± SEM of four independent experiments. Ro 31–8220 IC_50_ value is indicated at the top of the graph. (C) HEK-OCT1 cells were incubated with 1 μM DAPI for 5 min at 37°C, in the presence or absence of 50 μM verapamil or 2 μM Ro 31–8220. After washing with ice-cold PBS, accumulation of DAPI was determined by spectrofluorimetry as described in Materials and Methods. Data are expressed as percentage of DAPI accumulation in control cells and are the means ± SEM of four independent experiments. *, p < 0.05 when compared with untreated cells (ANOVA followed by Dunnett's post-hoc test). (D) HEK-OCT1 cells were either untreated or exposed to 50 μM verapamil or 10 μM Ro 31–8220 for 5 min. After washing, cells were re-incubated in drug-free culture medium for 1 h. Cells were next incubated with 40 μM [^14^C]TEA for 5 min at 37°C. Intracellular accumulation of labelled TEA was then determined by scintillation counting. Data are expressed as % of accumulation of TEA found in untreated control cells, set at 100%, and are the means ± SEM of three independent experiments. *, p < 0.05 when compared with untreated cells (ANOVA followed by Dunnett's post-hoc test).

To investigate the features of the PKC-independent inhibition of OCT1 activity by Ro 31–8220, we first analyzed the effects of the BIM on OCT1 localization in HEK-OCT1 cells, because rapid retrieval of transporters from plasma membrane is a well-established cause of down-regulation of transporter activity [42, 43]. As shown in S4 Fig, exposure to Ro 31–8220 failed to obviously alter plasma membrane expression of OCT1. We next determined whether the BIM may directly *cis*-inhibit OCT1 like verapamil and other OCT inhibitors. Simultaneous incubation with TEA and 2 μM Ro 31–8220 for a short time (5 min) resulted in a marked inhibition of TEA accumulation in HEK-OCT1 cells and also in human hepatoma HepaRG cells (Fig 3A), which constitutively exhibit OCT1 activity in an hepatic environment [30]. This *cis*-inhibition of OCT1-mediated TEA accumulation by Ro 31–8220 was found to be concentration-dependent, with an IC_50_ of 0.18 ± 0.05 μM (Fig 3B). In addition to uptake of TEA, that of the OCT1 substrate DAPI was found to be markedly inhibited by 2 μM Ro 31–8220 in HEK-OCT1 cells (Fig 3C); the reference OCT1 inhibitor verapamil similarly inhibited DAPI uptake. Finally, to test the putative reversibility of Ro 31-8220-mediated OCT1 inhibition, HEK-OCT1 cells were first incubated with Ro 31–8220 or verapamil for 5 min, washed and re-incubated in drug-free culture medium for 1 h, before analysis of OCT1-mediated TEA transport. As shown in Fig 3D, cells pre-incubated with Ro 31–8220 exhibited strong inhibition of OCT1-mediated TEA accumulation, in contrast to counterparts pre-incubated with verapamil, which displayed only marginal, although significant, inhibition of TEA uptake. Such data consequently indicate that OCT1 inhibition caused by Ro 31–8220 was not reversible, in contrast to that due to verapamil.

**Fig 4:**
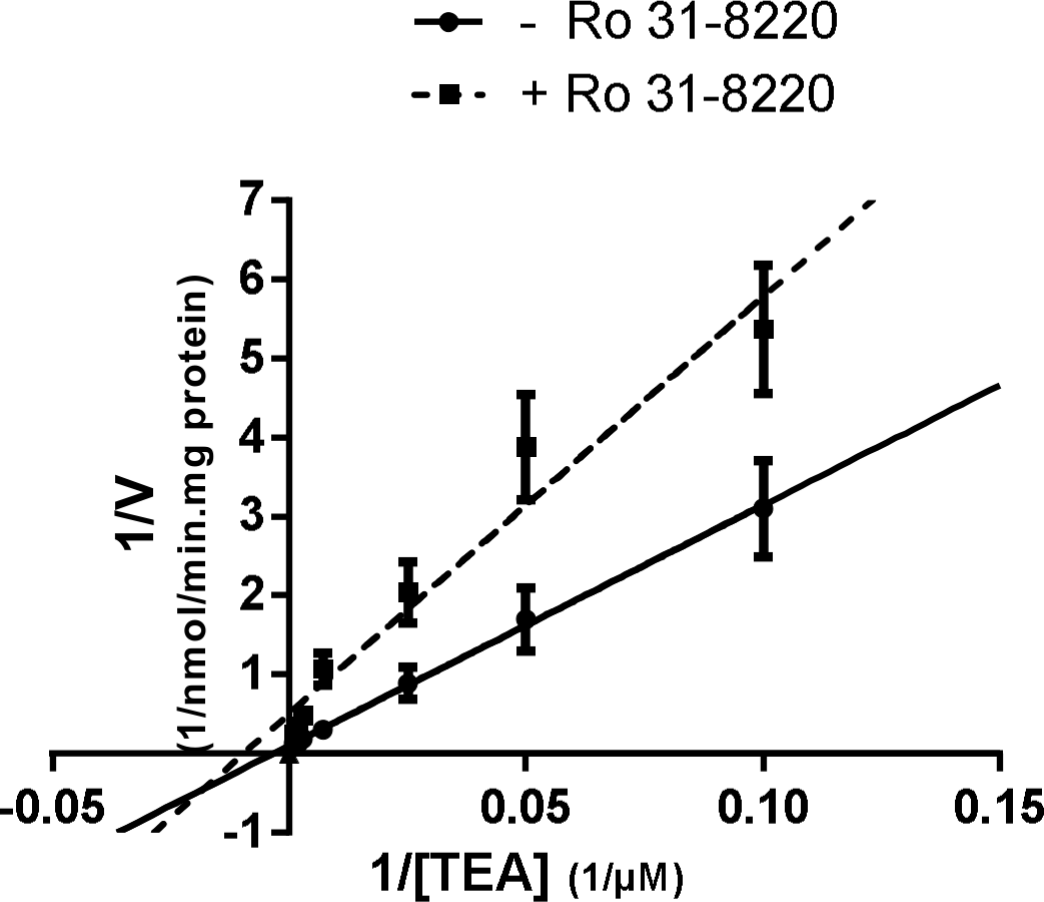
Lineweaver-Burk plot of the inhibitory effect of Ro 31–8220 on TEA uptake in HEK-OCT1 cells. HEK-OCT1 cells were incubated with various concentrations of TEA (from 10 to 1040 μM) for 5 min at 37°C, in the absence or presence of 0.5 μM Ro 31–8220. After washing with ice- cold PBS, intracellular accumulation of TEA was determined by scintillation counting and normalized to total protein content. Data shown are the means ± SEM of four independent experiments.

Mode of action for Ro 31-8220-mediated inhibition of OCT1 activity was next evaluated through graphical analysis of Lineweaver-Burke plots of 1/v versus 1/[TEA] in the presence or absence of the BIM. As shown in Fig 4, the plots were consistent with a mixed inhibition of OCT1 activity by Ro 31–8220, *i*.*e*., the intercept of the plots with or without the BIM was out of the x-axis and the y-axis.

**Fig 5:**
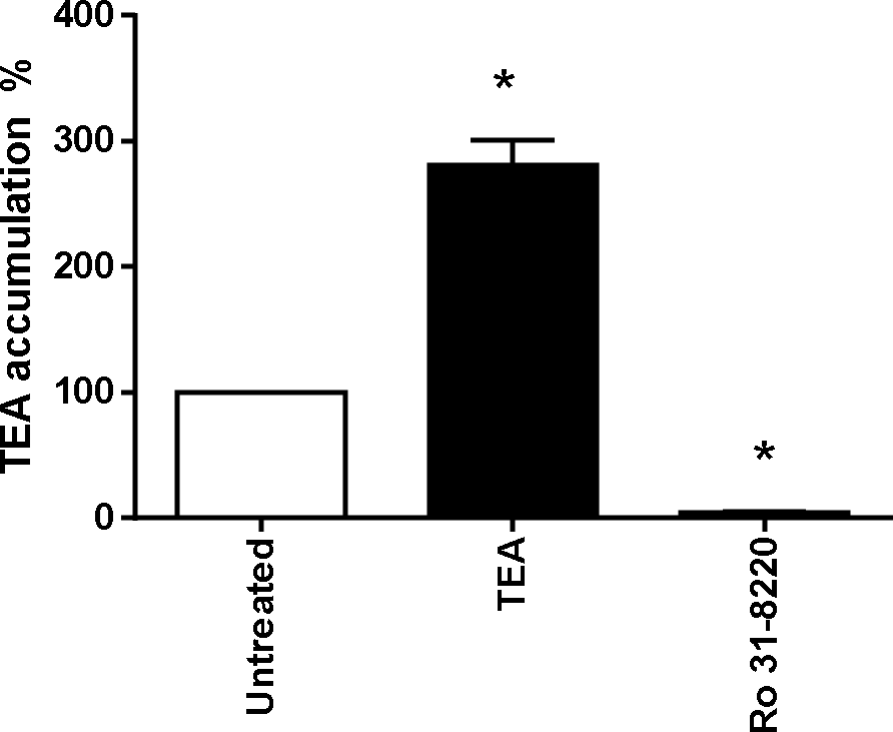
Trans-inhibition of OCT1 activity by Ro 31–8220. HEK-OCT1 cells were either untreated or exposed to 2 mM unlabelled TEA or 10 μM Ro 31–8220 for 1 h. After washing, cells were next incubated with 40 μM [^14^C]TEA for 5 min at 37°C. Intracellular accumulation of labelled TEA was then determined by scintillation counting. Data are expressed as % of accumulation of TEA found in untreated control cells, set at 100%, and are the means ± SEM of four independent experiments. *, p < 0.05 when compared with untreated cells (ANOVA followed by Dunnett's post-hoc test).

Finally, we investigated whether Ro 31–8220 can *trans*-stimulate TEA uptake, which may constitute an argument in favor of the transport of the BIM by OCT1 [34]. As shown in Fig 5, pre-loading with Ro 31–8220 resulted in *trans*-inhibition of radiolabeled TEA uptake. By contrast, pre-loading with unlabeled TEA led to a *trans*-stimulation of radiolabeled TEA uptake (Fig 5), as expected for an OCT1 substrate like TEA [34].

### Effect of various BIMs on OCT1 activity

**Fig 6:**
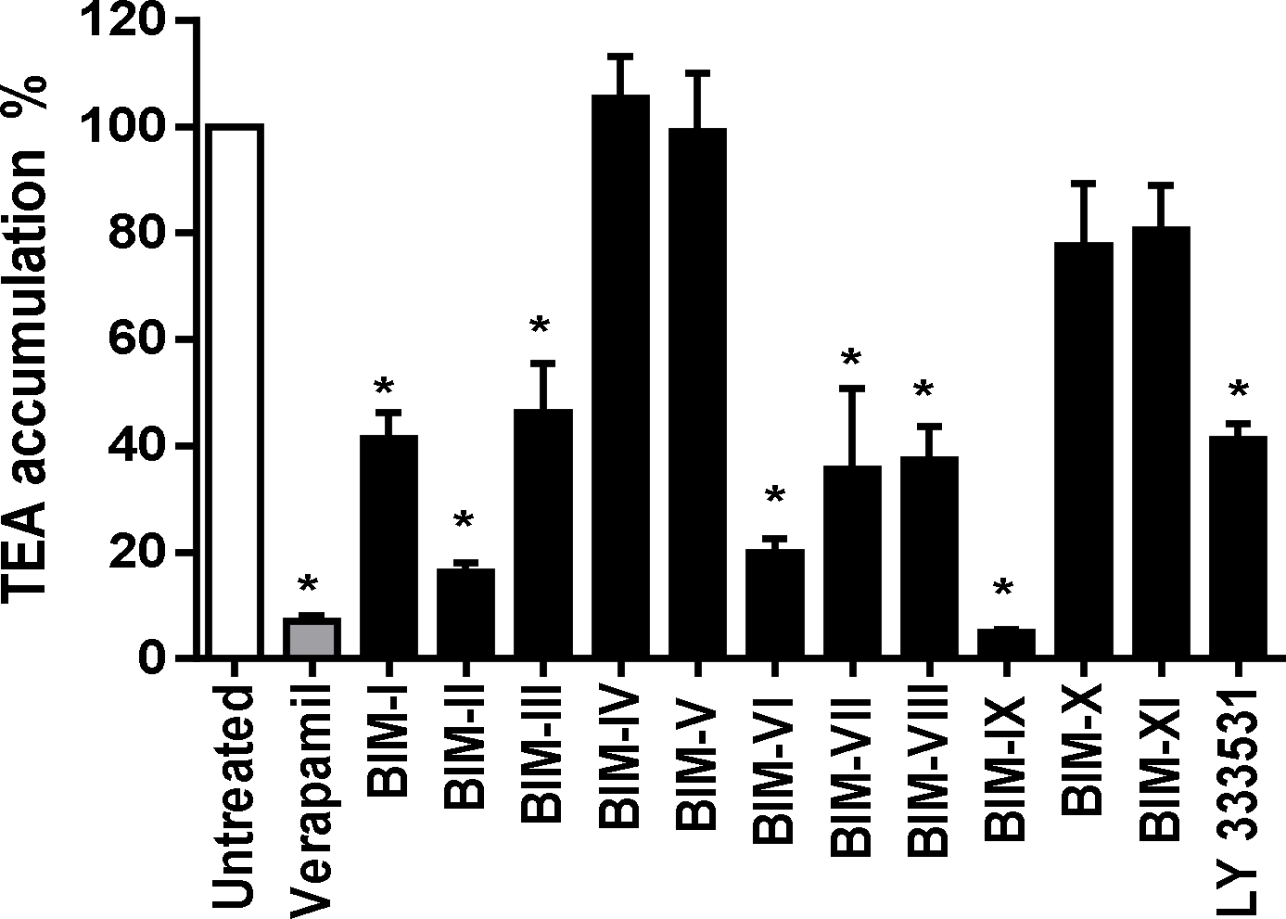
Inhibition of OCT1 activity by various BIM-related molecules. HEK-OCT1 cells were incubated with 40 μM [^14^C]-TEA for 5 min at 37°C in the presence or absence of 50 μM verapamil or of various BIMs, used each at 5 μM. After washing with ice-cold PBS, intracellular accumulation of [^14^C]-TEA was determined by scintillation counting. Data are expressed as % of accumulation of TEA found in control cells, set at 100%, and are the means ± SEM of three independent experiments. *, p < 0.05 when compared with untreated cells (ANOVA followed by Dunnett's post-hoc test).

To determine whether the inhibitory effect of Ro 31–8220 towards OCT1 activity may be shared by structural analogs, the effects of various BIMs and of the PKC inhibitor LY 333531, which also contains a BIM core (Fig 1), on OCT1-mediated transport were studied. In addition to Ro 31–8220, several BIMs such as BIM-I, BIM-II, BIM-III, BIM-VI, BIM-VII, BIM-VIII (also known as Ro 31–7549) and LY 333531 significantly inhibited OCT1-mediated TEA accumulation in HEK-OCT1 cells by more than 50% when compared to control TEA uptake (Fig 6). By contrast, BIM-IV (also known as Arcyriarubin A), BIM-V, BIM-X (also known as Ro 31–8425) and BIM-XI (also known as Ro 31–0432) failed to significantly alter TEA accumulation (Fig 6).

In order to identify the specific physico-chemical properties associated to OCT1 inhibition for BIMs, molecular descriptors, including 0D-constitutional, 1D-structural, 2D-topological and 3D-geometrical descriptors, were determined using Dragon 6 software. Putative correlations with OCT1 activity inhibition were next analyzed using Pearson’s correlation test. Using a cutoff of |r| > 0.8, 209 molecular descriptors were found to be correlated with inhibition of OCT1 activity; the correlation was positive (r > 0.8) or negative (r < -0.8) for 170 and 37 descriptors, respectively (Table 1). These molecular descriptors associated with OCT1 inhibition belong to different blocks of descriptors, especially those of 2D-matrix descriptors (n = 82), of 3D-RDF (Radial Distribution Function) descriptors (n = 20), of 3D-WHIM descriptors (n = 25) and of 3D GETAWAY (Geometry, Topology and Atom-Weights Assembly) descriptors (n = 16) (Table 1). When a more stringent cut-off value of |r| > 0.9 was applied, 13 molecular descriptors were found to be correlated with OCT1 activity inhibition (Table 2); linear regression analysis indicated a highly significant positive linear relation between the index values of these descriptors and the % of OCT1 activity inhibition (S5 Fig). Most of these molecular descriptors belong to the 3D-WHIM category of descriptors and concern notably total size indexes unweighted (Au and Vu) or weighted by various parameters such as van der Walls volume (Av and Vv), polarizability (Ap and Vp) or ionization potential (Ai and Vi) (Table 2).

**Table 1:** Repartition of molecular descriptors correlated with OCT1 activity inhibition for BIMs (|r|>0.80).

Dragon ID Block	Block category	Block description	Number of descriptors	Number of correlated descriptors		% of correlated descriptors in each block
				Positive correlation (r > 0.8)	Negative correlation (r < -0.8)	
**1**	0D-descriptors	Constitutional descriptors	43	0	0	0.0
**2**	0D-descriptors	Ring descriptors	32	0	0	0.0
**3**	2D-descriptors	Topological indices	75	11	0	14.7
**4**	2D-descriptors	Walk and path counts	46	0	0	0.0
**5**	2D-descriptors	Connectivity indices	37	1	0	2.7
**6**	2D-descriptors	Information indices	48	3	0	6.3
**7**	2D-descriptors	2D matrix-based descriptors	550	82	7	16.2
**8**	2D-descriptors	2D autocorrelations	213	2	0	0.9
**9**	2D-descriptors	Burden eigenvalues	96	0	0	0.0
**10**	2D-descriptors	P_VSA-like descriptors	45	0	0	0.0
**11**	2D-descriptors	ETA indices	23	0	0	0.0
**12**	2D-descriptors	Edge adjacency indices	324	0	0	0.0
**13**	3D-descriptors	Geometrical descriptors	38	3	0	7.9
**14**	3D-descriptors	3D matrix-based descriptors	90	4	4	8.9
**15**	3D-descriptors	3D autocorrelations	80	5	9	17.5
**16**	3D-descriptors	RDF descriptors	210	20	0	9.5
**17**	3D-descriptors	3D-MoRSE descriptors	224	6	0	2.2
**18**	3D-descriptors	WHIM descriptors	114	25	0	21.9
**19**	3D-descriptors	GETAWAY descriptors	273	0	16	5.9
**20**	3D-descriptors	Randic molecular profiles	41	5	0	12.2
**21**	0D-descriptors	Functional group counts	154	0	0	0.0
**22**	1D-descriptors	Atom-centred fragments	115	0	0	0.0
**23**	1D-descriptors	Atom-type E-state indices	170	0	0	0.0
**24**	2D-descriptors	CATS 2D	150	0	0	0.0
**25**	2D-descriptors	2D Atom Pairs	1596	3	0	0.1
**26**	3D-descriptors	3D Atom Pairs	36	0	0	0.0
**27**	others	Charge descriptors	15	2	1	20.0
**28**	others	Molecular properties	20	0	0	0.0
**29**	others	Drug-like indices	27	0	0	0.0

**Table 2:** Molecular descriptors highly correlated with OCT1 activity inhibition for BIMs (r>0.90).

Block description	Descriptor ID	Significance	r	p-value
**3D-MoRSE descriptors**	Mor02s	Signal 02 / weighted by I-stat	0.9297	< 0.0001
**3D-WHIM descriptors**	Au	A total size index / unweighted	0.9462	< 0.0001
	Am	A total size index / weighted by mass	0.9437	< 0.0001
	Av	A total size index / weighted by van der Waals volume	0.9463	< 0.0001
	Ae	A total size index / weighted by Sanderson electronegativity	0.9505	< 0.0001
	Ap	A total size index / weighted by polarizability	0.9382	< 0.0001
	Ai	A total size index / weighted by ionization potential	0.9488	< 0.0001
	As	A total size index / weighted by I-stat	0.9241	< 0.0001
	Vu	V total size index / unweighted	0.9131	< 0.0001
	Vv	V total size index / weighted by van der Waals volume	0.9079	< 0.0001
	Vp	V total size index/ weighted by polarizability	0.9226	< 0.0001
	Vi	V total size index / weighted by ionization potential	0.9038	< 0.0001
**2D atom pairs**	F10[C-N]	Frequency of C—N at topological distance 10	0.9123	<0.0001

### Effect of Ro 31–8220 on OCT2, MATE1 and MATE2-K activities

**Fig 7:**
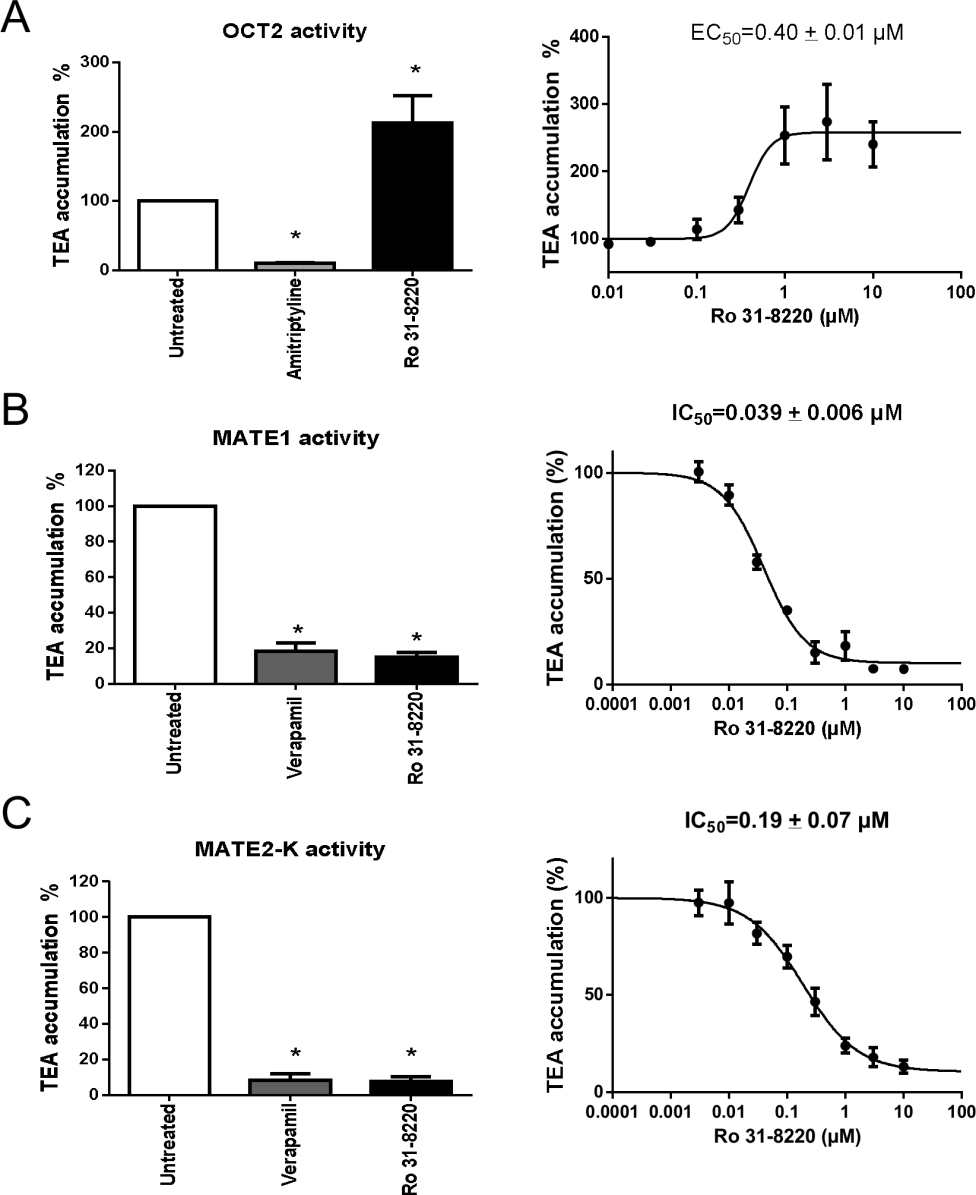
Effect of Ro 31–8220 on OCT2, MATE1 and MATE2-K activities. (A) HEK-OCT2 cells were incubated with 40 μM [^14^C]-TEA for 5 min at 37°C and pH = 7.4, in the presence or absence of (Left) 500 μM amitriptyline or 5 μM Ro 31–8220, or of (Right) various concentrations (from 0.01 to 10 μM) of Ro 31–8220. After washing by ice-cold PBS, intracellular accumulation of TEA was determined by scintillation counting. Data are expressed as % of accumulation of TEA found in control cells, set at 100%, and are the means ± SEM of three independent experiments. (Left) *, p < 0.05 when compared with untreated cells (ANOVA followed by Dunnett's post-hoc test); (Right) Ro 31–8220 EC_50_ value towards OCT2 activity is indicated at the top of the graph. (B) HEK-MATE1 or (C) HEK-MATE2-K cells were incubated with 40 μM [^14^C]-TEA for 5 min at 37°C and pH = 8.4, in the presence or absence of (Left) 200 μM verapamil or 5 μM Ro 31–8220, or of (Right) various concentrations (from 0.003 to 10 μM) of Ro 31–8220. After washing by ice-cold PBS, intracellular accumulation of TEA was determined by scintillation counting. Data are expressed as % of accumulation of TEA found in control cells, set at 100%, and are the means ± SEM of three independent experiments. (Left) *, p < 0.05 when compared with untreated cells (ANOVA followed by Dunnett's post-hoc test); (Right) Ro 31–8220 IC_50_ values towards (B) MATE1 and (C) MATE2-K activities are indicated at the top of the graphs.

The effects of Ro 31–8220 on activity of organic cation transporters sharing functional features with OCT1 such as OCT2, MATE1 and MATE2-K [44] were finally determined. For this purpose, we used HEK-OCT2 cells, exhibiting high level of amitriptyline-inhibitable uptake of TEA when compared to HEK-MOCK cells (S1B Fig) and HEK-MATE1 and HEK-MATE2-K cells displaying high level of verapamil-inhibitable uptake of TEA comparatively to HEK-MOCK cells (S1C Fig). Surprisingly, Ro 31–8220 used at 5 μM, in contrast to the reference OCT2 inhibitor amitriptyline, was found to enhance TEA accumulation in HEK-OCT2 cells (Fig 7A, Left). This effect was concentration-dependent, with an EC_50_ of 0.40 ± 0.01 μM (Fig 7A, Right). Determination of kinetic parameters (K_m_ and V_max_) of OCT2-mediated accumulation of TEA in the absence or presence of Ro 31–8220 next revealed that the BIM significantly decreased K_m_ value by a 2.8-fold-factor (S6 Fig). By contrast, Ro 31–8220 failed to enhance accumulation of TEA in HEK-MOCK cells (data not shown). Ro 31–8220 was next found to inhibit TEA accumulation in both HEK-MATE1 and HEK-MATE2-K cells (Fig 7B, Left and Fig 7C, Left). Such effects, also observed with the reference MATE inhibitor verapamil, were concentration-dependent, with Ro 31–8220 IC_50_ of 0.039 ± 0.006 μM and 0.19 ± 0.07 μM for MATE1 and MATE2-K, respectively (Fig 7B, Right and Fig 7C, Right).

## Discussion

Various off-targets have already been described for the potent PKC inhibitor Ro 31–8220, including blockage of the ABC transporter BCRP [25]. The data reported in the present study extend these PKC-independent effects of the BIM to organic cation transporters, especially OCT1. Indeed, Ro 31–8220 was found to block transport of the reference OCT1 substrates TEA and DAPI in HEK-OCT1 cells. This inhibition of OCT1 activity by the BIM was not observed in response to BIM-independent PKC inhibitors such as staurosporine and chelerythrine; moreover, some BIMs known to inhibit PKCs such as BIM-IV, BIM-X and BIM-XI [1, 4] also failed to inhibit OCT1 activity, thus fully indicating that Ro 31-8220-mediated inhibition of OCT1 activity is not dependent on PKC inhibition. In addition, the fact that activation of PKCs by the phorbol ester PMA failed to regulate OCT1 activity demonstrates a PKC-independent functionality of the OCT1 transporter, which does not argue in favor of PKC inhibition involvement in Ro 31–8220 effects towards OCT1 activity.

The PKC-independent mechanism by which Ro 31–8220 inhibits OCT1 activity remains to be determined. An unspecific and general inhibitory effect of Ro 31–8220 towards SLC transporters can be discarded because (i) this compound failed to alter activity of the bile salt transporter NTCP and (ii) it stimulated, and did not inhibit, OCT2 activity. An effect of Ro 31–8220 towards expression level of OCT1 at the membrane is similarly very unlikely to contribute to inhibition of OCT1 activity because the BIM failed to alter membrane location of the transporter, as demonstrated by immunocytochemistry studies. In fact, Ro 31–8220 most likely inhibits OCT1 by directly binding to the transporter, as usually described for inhibitors of ABC and SLC transporters [45]. The inhibition of OCT1 activity by the PKC inhibitor was moreover not reversible, in contrast to that due to verapamil, thus suggesting that Ro 31–8220 binding to OCT1 is irreversible. Interestingly, analysis of the mechanism of inhibition using Lineweaver-Burke plots suggests a mixed inhibition of OCT1 activity by Ro 31–8220. This type of inhibition is mostly allosteric in nature, where the inhibitor binds to a site other than the active site to cause a conformational change in the transporter structure, reducing the affinity of substrate for the active site. The fact that Ro 31–8220 is not a substrate for OCT1, as demonstrated by the absence of *trans*-stimulation effect, also supports a binding of the BIM outside the active site of transport. The presence of binding sites for inhibitors that may be distinct from the active site remain however to be formally determined for human OCT1. By contrast, for rat Oct1, both low affinity substrate binding sites and high affinity substrate binding sites, that can mediate inhibition via non-transported compounds, have been reported [46].

Besides Ro 31–8220, several other BIMs blocked OCT1 activity. Various molecular descriptors correlated with % of inhibition of OCT1 activity by these BIMs. These descriptors belong to different blocks of 2D-topological and 3D-geometrical descriptors, indicating that various physico-chemical parameters are likely to contribute to OCT1 inhibition by BIMs. Some 3D-WHIM descriptors corresponding to total size indexes of the 3D molecular structure of BIMs, unweighted or weighted by van der Wals volume, polarizability or ionization potential, were found to be highly correlated with inhibition of OCT1 activity by BIMs and to exhibit a linear relation with % of OCT1 activity inhibition. This suggests that these 3D-WHIM descriptors correspond to crucial molecular descriptors, that may have to be priority considered for OCT1-mediated transport blockage. Interestingly, OCT1 inhibition may represent a promising way for treatment of hepatic steatosis such as non-alcoholic fatty liver disease, through inhibition of OCT1-mediated thiamine transport and subsequent activation of the energy sensor AMP-activated kinase [27]. Further studies are therefore likely required to confirm the relevance of 3D-WHIM descriptors for getting potent OCT1 inhibitors and, beyond, to more accurately determine the physico-chemical parameters important for blocking OCT1 activity.

In addition to OCT1, other organic cation transporters such as OCT2, MATE1 and MATE2-K were also impacted by Ro 31–8220. Indeed, the BIM markedly inhibited MATE1- and MATE2-K-mediated transport of TEA, with low IC_50_ values, *i*.*e*., in the 0.01–0.1 μM range, which clearly positions it as a potent inhibitor of MATE1 and MATE-2K activity. These data therefore confirm that OCT1, MATE1 and MATE2-K, with share common substrates such as TEA and metformin, also share common inhibitors such as Ro 31–8220. Various compounds such as pentimidine or mitoxantrone have also been demonstrated to inhibit these three SLC transporters, whereas other inhibitors such as ondansetron more specifically block MATE1 and MATE2-K or even target only MATE1 such as pantoprazole [47]. Additional studies are likely required to better understand the molecular basis of these differential patterns of inhibition of organic cation transporters by various chemicals. With respect to OCT2 activity, it was unexpectedly *cis*-stimulated by Ro 31–8220. This effect was concentration-dependent, with an EC_50_ value (0.40 μM) close to IC_50_ values for OCT1, MATE1 and MATE2-K inhibition, indicating that the same range of Ro 31–8220 concentrations either inhibits OCT1, MATE1 and MATE2-K or stimulates activity of OCT2. Ro 31–8220 likely cis-stimulated OCT2-mediated transport of TEA through enhancing the affinity of TEA to OCT2, as suggested by the decrease of the kinetic parameter K_m_ in response to the BIM. *Cis*-stimulation of transporter activity by chemicals is rather poorly documented for OCT2 in the literature, but has been described for other transporters, notably MRP2 (*ABCC2*) [48], organic anion-transporting polypeptide (OATP) 1B1 (*SLCO1B1*) and OATP1B3 (*SLCO1B3*) [49, 50]. The exact mechanism involved in such stimulation of transporter activities remains to be determined. For MRP2, it may be linked to the presence of two similar but non-identical ligand binding sites: one site from which substrate is transported and a second site that regulates the affinity of the transport site for the substrate, and to which stimulators may bind [51]. Further studies are required to determine whether OCT2 stimulation by Ro 31–8220 also requires a regulatory binding site.

The interactions of Ro 31–8220 with organic cation transporters have likely to be kept in mind when using this BIM as a pan-PKC inhibitor in experimental studies. Indeed, PKC inhibition in response to Ro 31–8220 is usually achieved with Ro 31–8220 concentrations ranging from 1 to 10 μM [52, 53], which are also highly effective towards OCT1, OCT2, MATE1 and MATE2-K, thus precluding the use of Ro 31–8220 or other BIMs for specifically analyzing putative PKC-dependent regulation of these organic cation transporters. Moreover, the use of Ro 31–8220 with cells expressing at least one of the targeted organic cation transporters is likely to inhibit transport of endogenous compound substrates for these transporters such as catecholamines (handled by OCT1 and OCT2) [54] or thiamine (handled by OCT1) [27], which may result in phenotypic effects irrelevant from PKC inhibition. Interactions of BIMs with organic cation transporters have also to deserve attention with respect to the potential clinical use of BIMs. Indeed, these compounds, such as LY 333531, have entered or are susceptible to enter clinical trials for the treatment of various diseases [55], including diabetic peripheral neuropathy [56], diabetic retinopathy [57] and cancers [58]. In this context, putative inhibition of organic cation transporters in BIMs-treated patients have likely to be considered owing to (i) potential drug-drug interactions through alteration of OCT/MATE-related pharmacokinetics features of co-administrated drugs and (ii) potential adverse effects due to inhibition of OCT/MATE-mediated transport of endogenous substrates. Additional studies are therefore required to determine whether Ro 31–8220 and other BIMs may inhibit *in vivo* activity of OCT/MATE and to characterize putative pharmacokinetics relevance.

In summary, the nature of cellular off-targets of the PKC inhibitor Ro 31–8220 and of other BIMs-related molecules was extended to organic cation transporters, especially OCT1. Such PKC-independent alterations of organic cation transport have likely to be kept in mind when using Ro 31–8220 and other BIMs as PKC inhibitors in experimental or clinical studies.

## Supporting Information

S1 Fig Accumulation of TEA in HEK-MOCK, HEK-OCT1, HEK-OCT2, HEK-MATE1 and HEK-MATE2-K cells. (A) HEK-MOCK and HEK-OCT1 cells, (B) HEK-MOCK and HEK-OCT2 cells and (C) HEK-MOCK, HEK-MATE1 and HEK-MATE2-K cells were incubated with 40 μM [^14^C]-TEA for 5 min at 37°C in the presence or absence of reference transporter inhibitors, *i*.*e*., (A) 50 μM verapamil, (B) 500 μM amitriptyline or (C) 200 μM verapamil, at indicated pH values. After washing with ice-cold PBS, intracellular accumulation of TEA was determined by scintillation counting and normalized to total protein content. Data are the means ± SEM of at least three independent experiments.*, p<0.05 when compared to HEK-MOCK cells (Student's *t*-test); #, p<0.05 when compared to cells not exposed to reference transporter inhibitor (Student's *t*-test).
(TIF)

S2 Fig Effect of Ro 31–8220 on NTCP activity. HEK-NTCP cells were either untreated or exposed to 2 μM Ro 31–8220 for 1 h. Cells were then incubated with 43.4 nM [^3^H]-taurocholate for 5 min at 37°C in the presence or absence of 100 μM cyclosporin A, used here as a reference NTCP inhibitor. After washing with ice-cold PBS, intracellular accumulation of taurocholate was determined by scintillation counting. Data are expressed as % of accumulation of taurocholate in untreated control cells, set at 100%, and are the means ± SEM of at least three independent experiments. *, p < 0.05 when compared with untreated cells (ANOVA followed by Dunnett's post-hoc test).
(TIF)

S3 Fig Inhibition of PMA-mediated ERK activation by staurosporine. HEK-OCT1 cells were untreated, treated with 100 nM PMA or 1 μM staurosporine or co-treated with PMA and staurosporine for 1 h. Phospho-ERK and total ERK protein contents were then determined by Western-blot analysis as described in Materials and Methods. Data shown are representative of two independent experiments.
(TIF)

S4 Fig Lack of effect of Ro 31–8220 on OCT1 localization. HEK-OCT1 cells were either untreated or exposed to 2 μM Ro 31–8220 for 1 h. Cells were next immunolabeled with monoclonal antibodies directed against OCT1 as described in Materials and Methods. Data shown are representative of two independent experiments. OCT1-related membrane staining is indicated by white arrows. Bar = 10 μm.
(TIFF)

S5 Fig Linear regression analysis of % of OCT1 activity inhibition for BIMs versus molecular descriptor values. Liner regression analysis was performed for the % of OCT1 activity inhibition versus values of 3D-WHIM descriptors Au, Am, Av, Ae, Ap, Ai, As, Vu, Vv, Vp and Vi, of the 3D-MoRSE descriptor Mor02s and of the 2D-atom pairs descriptor F10[C-N] for BIMs (n = 12). The r^2^ value, a measure of the goodness of the fit, and the p-value are indicated at the top of the graphs.
(TIF)

S6 Fig Effects of Ro 31–8220 on kinetic parameters of TEA accumulation in HEK-OCT2 cells. HEK-OCT2 cells were incubated with increasing concentrations of TEA in the absence or presence of 10 μM Ro 31–8220 for 5 min. TEA uptake velocity (v) was next fitted to [TEA] according to the Michaelis-Menten equation, in order to determine K_m_ and V_max_ values (indicated at the top of the graph). Each value shown is the mean ± S.E.M. of four independent experiments.*, p<0.05 when compared to cells not exposed to Ro 31–8220 (F-test).
(TIF)
