# Supplementary figures and images for: Protein Kinase C-Independent Inhibition of Organic Cation Transporter 1 Activity by the Bisindolylmaleimide Ro 31-8220
Source: PLoS One. 2015 Dec 10;10(12):e0144667. doi: 10.1371/journal.pone.0144667 (PMC4675551; doi:10.1371/journal.pone.0144667)

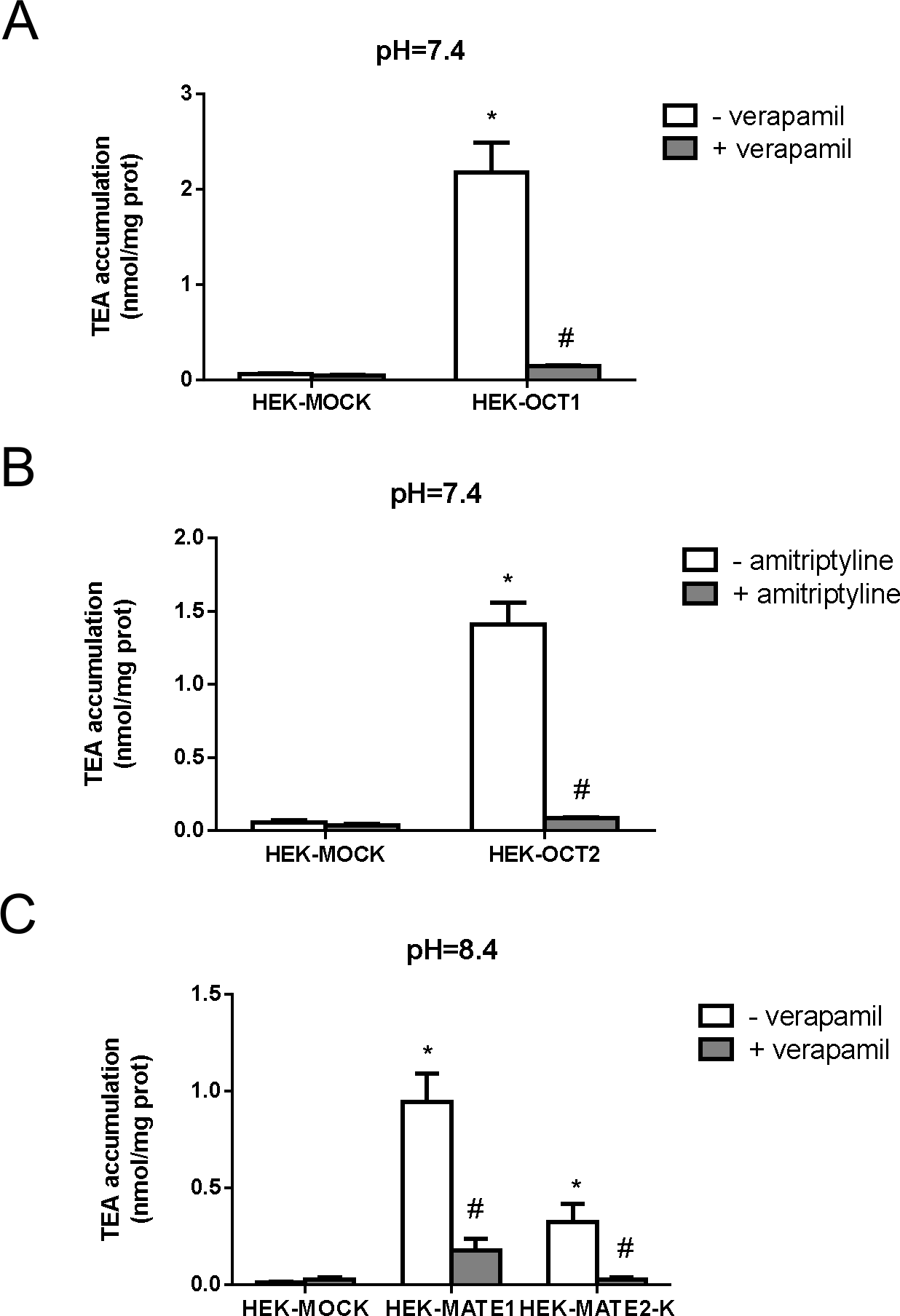

Supplement: S1 Fig — (A) HEK-MOCK and HEK-OCT1 cells, (B) HEK-MOCK and HEK-OCT2 cells and (C) HEK-MOCK, HEK-MATE1 and HEK-MATE2-K cells were incubated with 40 μM [14C]-TEA for 5 min at 37°C in the presence or absence of reference transporter inhibitors, i.e., (A) 50 μM verapamil, (B) 500 μM amitriptyline or (C) 200 μM verapamil, at indicated pH values. After washing with ice-cold PBS, intracellular accumulation of TEA was determined by scintillation counting and normalized to total protein content. Data are the means ± SEM of at least three independent experiments.*, p<0.05 when compared to HEK-MOCK cells (Student's t-test); #, p<0.05 when compared to cells not exposed to reference transporter inhibitor (Student's t-test). (TIF) [file pone.0144667.s001.tif]

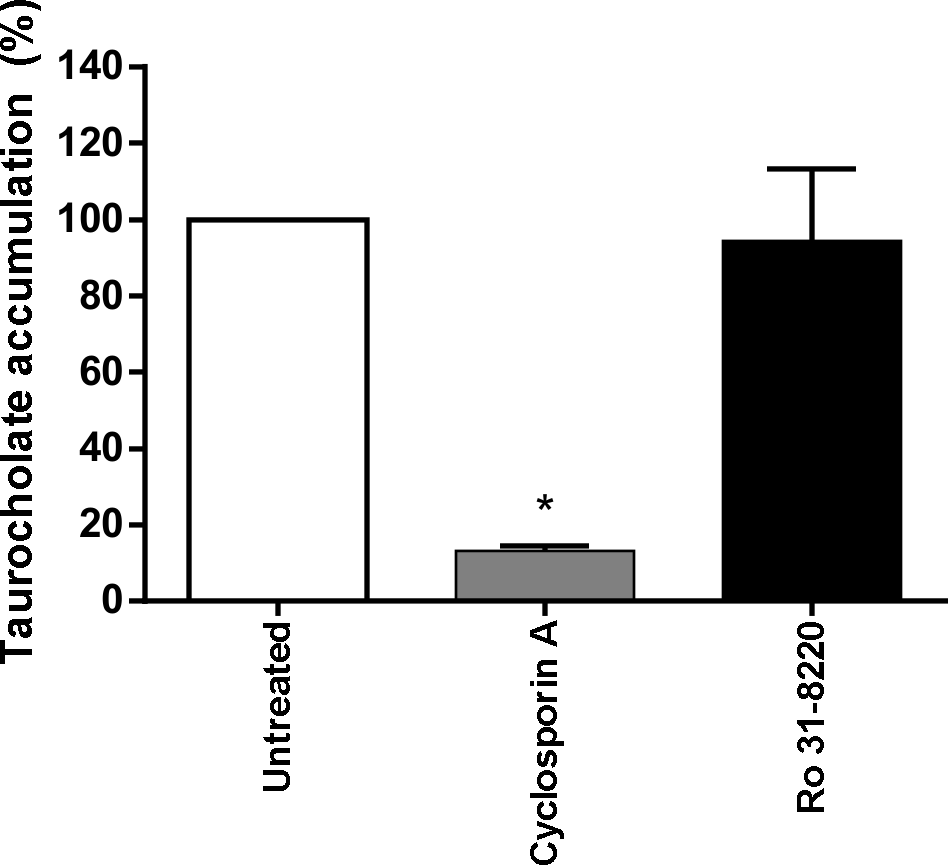

Supplement: S2 Fig — HEK-NTCP cells were either untreated or exposed to 2 μM Ro 31–8220 for 1 h. Cells were then incubated with 43.4 nM [3H]-taurocholate for 5 min at 37°C in the presence or absence of 100 μM cyclosporin A, used here as a reference NTCP inhibitor. After washing with ice-cold PBS, intracellular accumulation of taurocholate was determined by scintillation counting. Data are expressed as % of accumulation of taurocholate in untreated control cells, set at 100%, and are the means ± SEM of at least three independent experiments. *, p < 0.05 when compared with untreated cells (ANOVA followed by Dunnett's post-hoc test). (TIF) [file pone.0144667.s002.tif]

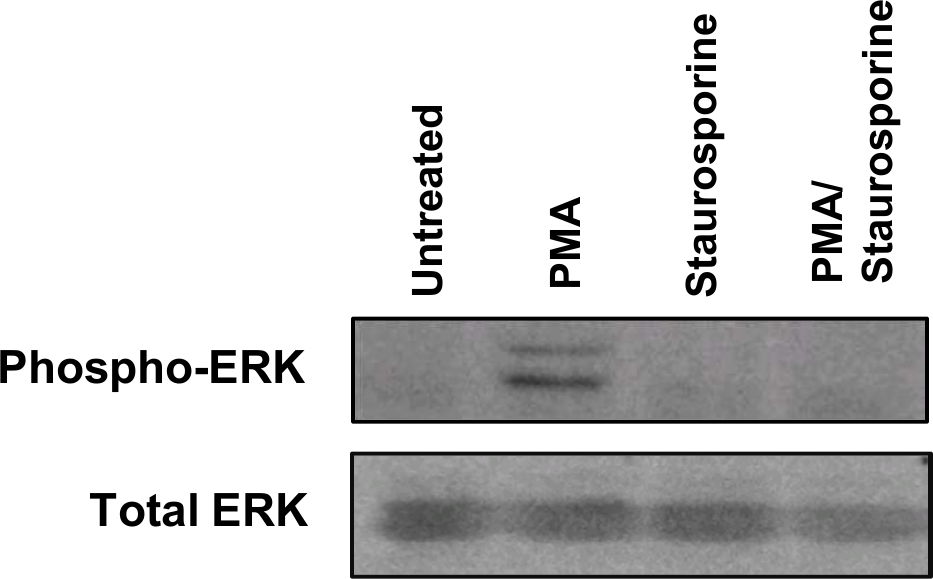

Supplement: S3 Fig — HEK-OCT1 cells were untreated, treated with 100 nM PMA or 1 μM staurosporine or co-treated with PMA and staurosporine for 1 h. Phospho-ERK and total ERK protein contents were then determined by Western-blot analysis as described in Materials and Methods. Data shown are representative of two independent experiments. (TIF) [file pone.0144667.s003.tif]

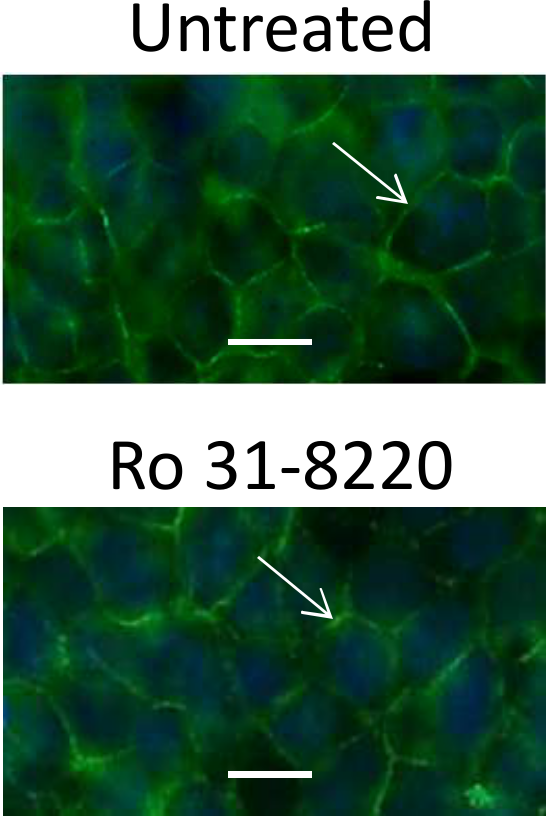

Supplement: S4 Fig — HEK-OCT1 cells were either untreated or exposed to 2 μM Ro 31–8220 for 1 h. Cells were next immunolabeled with monoclonal antibodies directed against OCT1 as described in Materials and Methods. Data shown are representative of two independent experiments. OCT1-related membrane staining is indicated by white arrows. Bar = 10 μm. (TIFF) [file pone.0144667.s004.tiff]

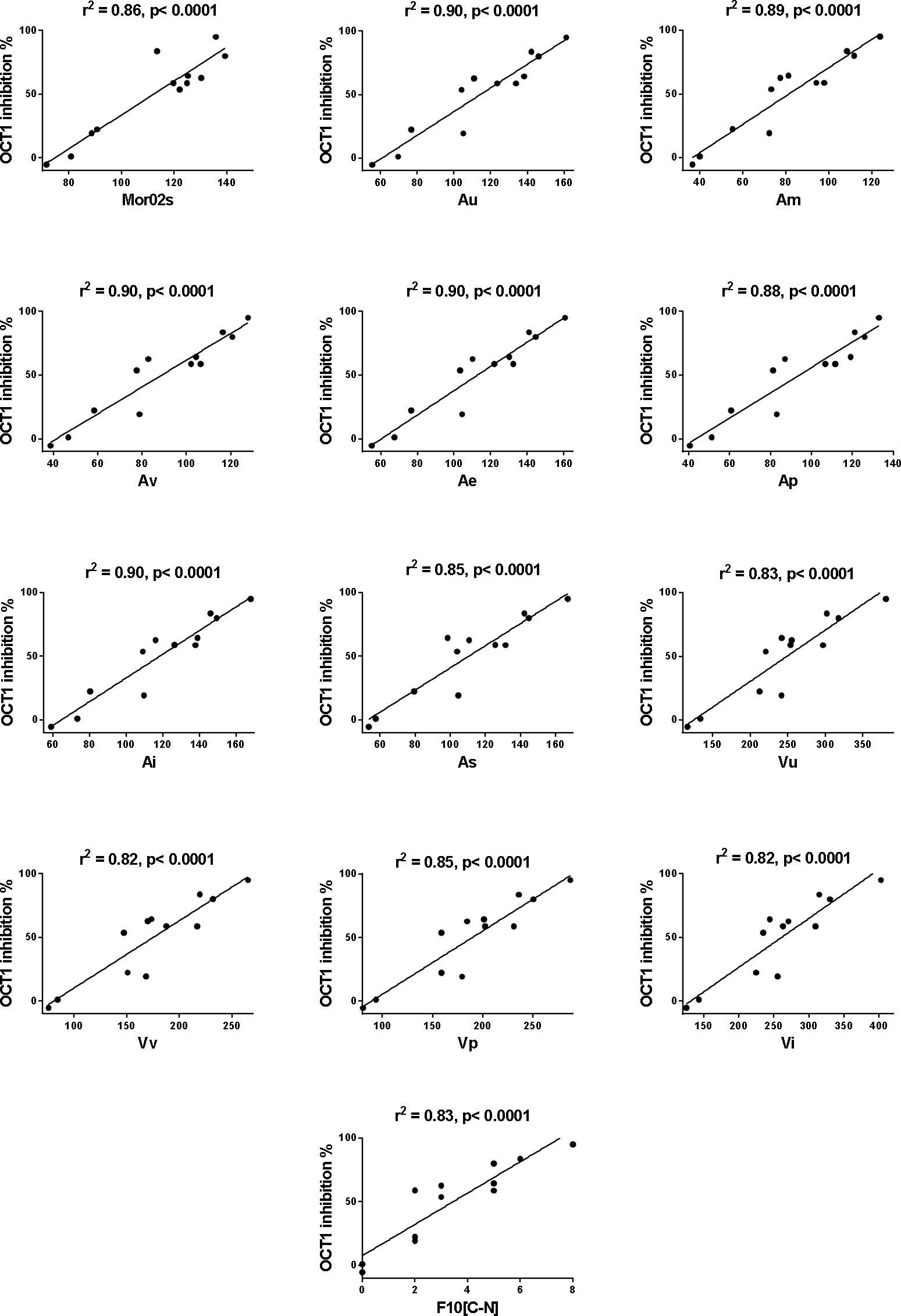

Supplement: S5 Fig — Liner regression analysis was performed for the % of OCT1 activity inhibition versus values of 3D-WHIM descriptors Au, Am, Av, Ae, Ap, Ai, As, Vu, Vv, Vp and Vi, of the 3D-MoRSE descriptor Mor02s and of the 2D-atom pairs descriptor F10[C-N] for BIMs (n = 12). The r2 value, a measure of the goodness of the fit, and the p-value are indicated at the top of the graphs. (TIF) [file pone.0144667.s005.tif]

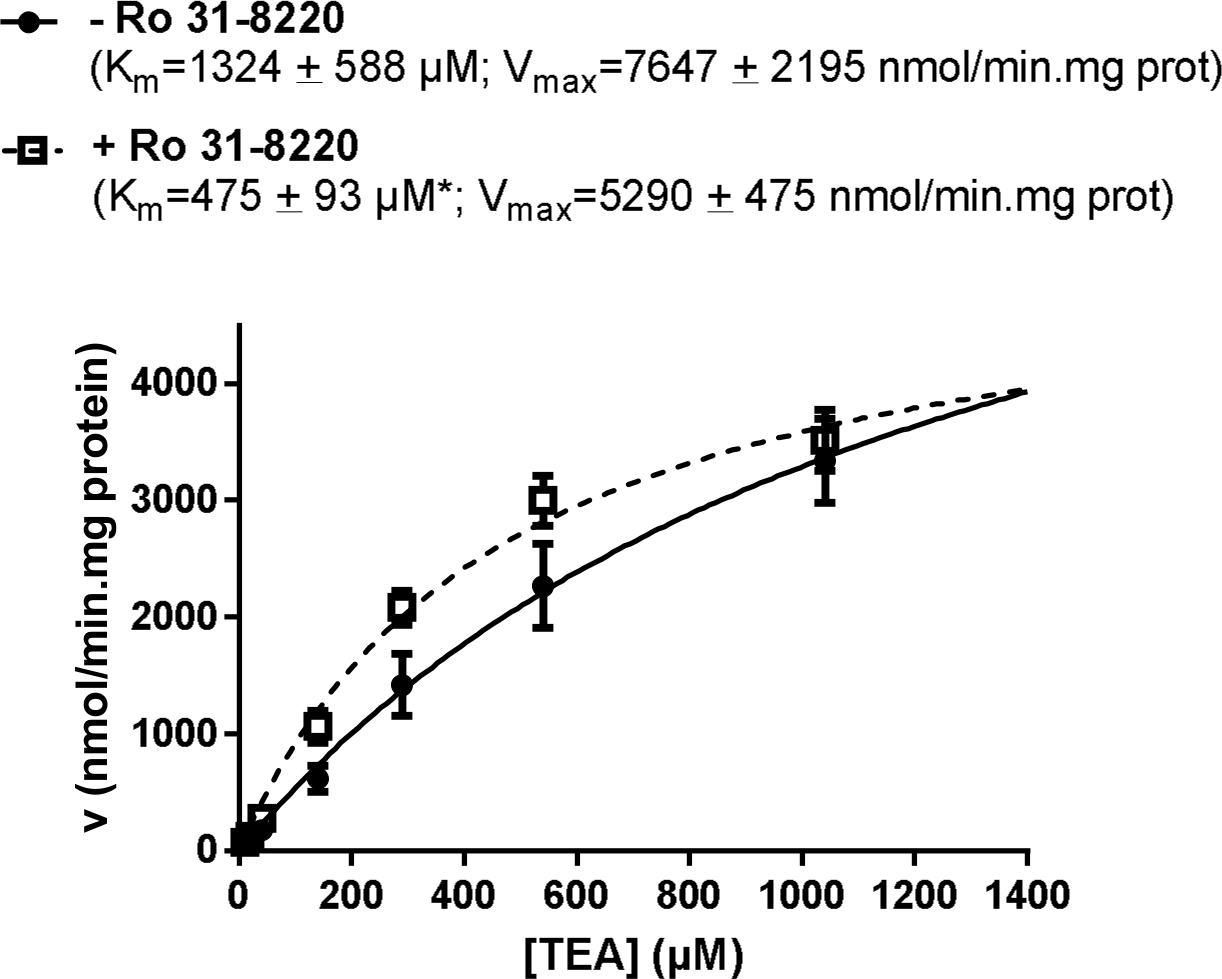

Supplement: S6 Fig — HEK-OCT2 cells were incubated with increasing concentrations of TEA in the absence or presence of 10 μM Ro 31–8220 for 5 min. TEA uptake velocity (v) was next fitted to [TEA] according to the Michaelis-Menten equation, in order to determine Km and Vmax values (indicated at the top of the graph). Each value shown is the mean ± S.E.M. of four independent experiments.*, p<0.05 when compared to cells not exposed to Ro 31–8220 (F-test). (TIF) [file pone.0144667.s006.tif]
